# Supplementary material for: Empirical methods for controlling false positives and estimating confidence in ChIP-Seq peaks
Source: BMC Bioinformatics. 2008 Dec 5;9:523. doi: 10.1186/1471-2105-9-523 (PMC2628906; doi:10.1186/1471-2105-9-523)
Supplement: Additional File 1 — A variety of html documents from the USeq web site detailing the available applications, their best usage, output file type descriptions, command line menus, etc. [file 1471-2105-9-523-S1.zip › USeqUserGuides/JavaDoc.zip!/JavaDoc/edu/utah/seq/data/Simulator.html]

Simulator


|  |  |  |
| --- | --- | --- |
| **Overview** \| **Package** \| **Class** \| **Use** \| **Tree** \| **Deprecated** \| **Index** \| **Help** | |  |
| **PREV CLASS**   **NEXT CLASS** | **FRAMES**    **NO FRAMES**     **All Classes** |  |
| SUMMARY: NESTED \| FIELD \| CONSTR \| METHOD | DETAIL: FIELD \| CONSTR \| METHOD |  |


---


## edu.utah.seq.data Class Simulator

```
java.lang.Object
  edu.utah.seq.data.Simulator
```

---

``` public class Simulator extends java.lang.Object ```

---

**Constructor Summary**

`Simulator(java.lang.String[] args)`
  
          Constructor


| **Method Summary** | |
| --- | --- |
| `java.lang.String` | `addError(java.lang.String read)`             Make sure your read length doesn't exceed the length of the error |
| `void` | `generateFragments()`             Pick points |
| `boolean` | `lotsOfNs(java.lang.String seq)`             Check N content. |
| `boolean` | `lotsOfRepeats(int minStart, int maxEnd)`             Checks repeat content |
| `static void` | `main(java.lang.String[] args)` |
| `void` | `pickRandomFragments(java.lang.String sequence, java.lang.String chromName, int numberPoints)`             Picks fragments and reads. |
| `int[]` | `selectHitsToChromosomes()`             Randomly picks chromosomes for generating points weighted by file size. |

**Methods inherited from class java.lang.Object**

`equals, getClass, hashCode, notify, notifyAll, toString, wait, wait, wait`

**Constructor Detail**

### Simulator

```
public Simulator(java.lang.String[] args)
```

:   Constructor


**Method Detail**

### selectHitsToChromosomes

```
public int[] selectHitsToChromosomes()
```

:   Randomly picks chromosomes for generating points weighted by file size.

---


### generateFragments

```
public void generateFragments()
```

:   Pick points

---


### lotsOfRepeats

```
public boolean lotsOfRepeats(int minStart,
                             int maxEnd)
```

:   Checks repeat content

---


### lotsOfNs

```
public boolean lotsOfNs(java.lang.String seq)
```

:   Check N content.

---


### pickRandomFragments

```
public void pickRandomFragments(java.lang.String sequence,
                                java.lang.String chromName,
                                int numberPoints)
```

:   Picks fragments and reads.

---


### addError

```
public java.lang.String addError(java.lang.String read)
```

:   Make sure your read length doesn't exceed the length of the error

---


### main

```
public static void main(java.lang.String[] args)
```


---


|  |  |  |
| --- | --- | --- |
| **Overview** \| **Package** \| **Class** \| **Use** \| **Tree** \| **Deprecated** \| **Index** \| **Help** | |  |
| **PREV CLASS**   **NEXT CLASS** | **FRAMES**    **NO FRAMES**     **All Classes** |  |
| SUMMARY: NESTED \| FIELD \| CONSTR \| METHOD | DETAIL: FIELD \| CONSTR \| METHOD |  |


---
